# Medical Items

**Published:** 1917-12

**Authors:** 


### MEDICAL ITEMS.

### THE GREAT AMERICAN HABIT.

"The great American habit" said a prominent physician a short time ago, "is neither rapid eating, nor dining 'too wisely and well.' These are evils, to be sure, but the one great habit to which most Americans are addicted is the routine use of laxatives and cathartics. Is it any wonder some European doctor has said we are a nation of constipationists! Why, physic has become only a little less necessary than the oxygen we breathe.

How true all this is, most physicians of broad experience will readily agree. Physic the first thing in the morning, physic before or after each meal, and physic the last thing at night! And only one purpose throughout, to move the bowels! Never a thought in regard to regulating them.

This is the pity of the whole situation, for instead of getting relief and correcting constipation, the average person is simply becoming more and more a slave to the cathartic habit.

To a certain extent this is not surprising for the great majority of remedies are simply "movers" not "regulators."

It was recognition of this fact that led to the preparation of Prunoids. Here is a remedy that is much more than a physic. Used as the physician's judgment will dictate, according to the needs of each patient. Prunoids will not only move the bowels satisfactorily without the least griping or disagreeable effect but will so stimulate the phsiologic processes of the intestinal tract that evacuations will become natural and regular.

It is the notable efficacy of Prunoids in correcting intestinal torpidity and restoring functional activity of the bowels that has led so many physicians to prefer this remedy to all others for the relief and permanent correction of chronic constipation. They have learned that the use of Prunoids offers a means not alone of correcting constipation, but what is especially gratifying, of curing "the great American habit."

"I prescribe Tongaline very frequently as a remedy for excess of uric acid, which is often the cause of rheumatism, and it is my sheet anchor for that condition. I also find Tongaline very beneficial in muscular pains, due to a sluggish liver and inactive bowels. When a patient comes to me complaining of soreness all over, I place him upon Tongaline, and it has never disappointed me."

"We think that all who have made a test of the action of Tongaline, either in the acute stage of grippe, or in the period of sonvalescense marked by an extreme nervous disturbance so often present, will be convinced that the remedy has a direct and marked influence for good.

There is not an organ or function in the body which may not be impaired or affected by grippe as to lead to a permanent disability, but on account of the extraordinary eliminative action of Tongaline, this rarely occurs if that remedy is used, since there is then no opportunity for such an accumulation of the poison as to induce permanent harm."

### POST-OPERATIVE QUIET.

For the purpose of relieving nervous irritability and bringing about rest following a serious operation, the surgeon has at his command in PASADYNE (Daniel) a sedative agent of the greatest usefulness and one free from evil effects. PASADYNE (Daniel) is merely a distinctive name for a pure concentrated tincture of passiflora incernate and is a product that has stood the test of nearly forty years. The advantage possessed by PASADYNE (Daniel) lie in its therapeutic potency and freedom from the properties that cause depression. Surgeons may depend upon it to secure for their patients the rest needed. A sample bottle of PASADYNE may be had by addressing the laboratory of John B. Daniel, Inc., Atlanta, Georgia.

